# Lesional Accumulation of CD163-Expressing Cells in the Gut of Patients with Inflammatory Bowel Disease

**DOI:** 10.1371/journal.pone.0069839

**Published:** 2013-07-26

**Authors:** Eleonora Franzè, Roberta Caruso, Carmine Stolfi, Massimiliano Sarra, Maria Laura Cupi, Flavio Caprioli, Ivan Monteleone, Francesca Zorzi, Daniela De Nitto, Alfredo Colantoni, Livia Biancone, Francesco Pallone, Giovanni Monteleone

**Affiliations:** 1 Department of Systems Medicine, University “TOR VERGATA” of Rome, Rome, Italy; 2 Unit of Gastroenterology and Pathology Unit, Fondazione Istituto di Ricovero e Cura a Carattere Scientifico Cà Granda Ospedale Maggiore Policlinico, Milan, Italy; 3 Department of Pathophysiology and Transplantation, University of Milan, Milan, Italy; University of Tor Vergata, Italy

## Abstract

Monocytes/macrophages displaying different markers of activation/differentiation infiltrate the inflamed gut of patients with inflammatory bowel diseases (IBD), but the role that each monocyte/macrophage subpopulation plays in the pathogenesis of IBD is not fully understood. The hemoglobin scavenger receptor CD163, a specific marker of monocytes/macrophages, has been associated with either anti-inflammatory or inflammatory functions of macrophages in several pathologies. In this study we examined the tissue distribution and function of CD163-expressing monocytes/macrophages in IBD. CD163 RNA and protein expression was more pronounced in IBD in comparison to normal controls, with no significant difference between Crohn's disease and Ulcerative colitis. In IBD, over-expression of CD163 was restricted to areas with active inflammation and not influenced by current therapy. Immunohistochemical analysis confirmed the accumulation of CD163-expressing cells in IBD, mostly around and inside blood vessels, thus suggesting that these cells are partly recruited from the systemic circulation. Indeed, FACS analysis of circulating mononuclear cells showed that the fractions of CD163-positive monocytes were increased in IBD patients as compared to controls. Functionally, interleukin-6 up-regulated CD163 expression in lamina propria mononuclear cells and mucosal explants of normal subjects. In IBD blood and mucosal cell cultures, cross-linking of CD163 with a specific monoclonal anti-CD163 antibody enhanced tumor necrosis factor-α synthesis. These findings indicate that IBD mucosa is abundantly infiltrated with CD163-positive cells, which could contribute to amplify the inflammatory cytokine response.

## Introduction

The gastrointestinal mucosa is the largest body surface to interface with the external environment and the largest reservoir of macrophages in the body [1], [2]. Preferentially located in the subepithelial lamina propria, intestinal macrophages play a key role in the maintenance of mucosal homeostasis and progression of tissue destructive immune responses. In the normal intestine, macrophages express low levels of microbe-associated molecular pattern receptors, including the lipopolysaccharide (LPS) receptors, CD14 and toll-like receptor (TLR)-4, produce anti-inflammatory molecules [e.g. (interleukin)-10, IL-25] and are able to engulf and kill microbes without eliciting inflammatory responses [3], [4], [5]. In contrast, during chronic inflammatory disorders, such as Crohn's disease (CD) and Ulcerative colitis (UC), the major forms of inflammatory bowel disease (IBD) in human beings, macrophages express high levels of CD14, TLRs and co-stimulatory molecules and synthesize huge amounts of pro-inflammatory mediators in response to bacterial stimulation [2], [6]. Phenotypic analysis of surface-cell markers has contributed to show that distinct macrophage subsets infiltrate the gut of patients with CD and patients with UC, even though the role that each macrophage subpopulation plays in the pathogenesis of IBD is not fully understood [7], [8].

The plasma membrane glycoprotein CD163, a member of the scavenger receptor cystein-rich (SRCR) superfamily class B, is highly expressed on resident tissue macrophages and to lesser extent on monocytes [9]. Initial studies identified CD163 as a scavenger receptor for hemoglobin (Hb)-haptoglobin (Hp) complexes formed during intravascular hemolysis [10]. More recently it has been demonstrated that CD163 can bind additional ligands, such as the tumor necrosis factor (TNF)-α-like weak inducer of the apoptosis protein, some bacteria and virus and either inhibit or amplify inflammatory responses depending on the context analyzed [11]. For example, in monocyte-derived macrophages treated with glucocorticoid, cross-linking of CD163 with EDHU1-Ab, a specific monoclonal anti-CD163 antibody direct against the SRCR3 domain of CD163, induces a protein tyrosine kinase- and casein kinase II-dependent signal that leads to calcium mobilization, inositol triphosphate production and secretion of pro-inflammatory cytokines (i.e. TNF-α, IL–1β and IL-6) [12], [13]. CD163 can also facilitate Gram-positive and -negative bacteria-induced proinflammatory cytokine response [14]. Consistently, up-regulation of CD163 has been documented in many inflammatory pathologies [15], [16], [17], [18]. On the other hand, there is evidence that CD163-expressing macrophages produce counter-regulatory molecules, which are involved in the resolution of many inflammatory processes [19]. In addition to the full-length moiety, a soluble form of CD163 (sCD163), generated by shedding of the cell-surface protein by matrix metalloproteinases, [20], [21] is elevated in many inflammatory conditions. sCD163 inhibits activation of human T lymphocytes thereby contributing to suppression of inflammatory responses [22].

Previous studies have evaluated CD163 in IBD with conflicting results [23], [24], [25] probably depending on the methodology adopted to assess the expression of the scavenger. Our study was aimed at further characterizing the tissue distribution of CD163 in IBD and ascertaining whether CD163-delivered signals are either inflammatory or anti-inflammatory in the gut. Using several techniques, we here show that: CD163-expressing cells are abundant in the inflamed intestine of patients with CD and patients with UC; IL-6, a cytokine over-produced in IBD, positively regulates CD163 expression in normal lamina propria mononuclear cells (LPMC) and mucosal explants; CD163 triggers inflammatory signals. Overall these data suggest a novel mechanism by which mucosal inflammation is amplified and perpetuated in IBD.

## Materials and Methods

### Ethics Statement

Each patient who took part in the study gave written informed consent and the study protocol was approved by the local Ethics Committees (Tor Vergata University Hospital, Rome).

### Patients and samples

Biopsies were taken from the inflamed mucosa of 12 patients with colonic CD, 7 patients with ileocolonic CD, 1 patient with ileal CD and 25 patients with UC undergoing colonoscopy for a clinically active disease at the Gastrointestinal Unit of Tor Vergata University (Rome, Italy) or Fondazione IRCCS Cà Granda, Ospedale Maggiore Policlinico (Milan, Italy). Paired biopsies were also taken from the inflamed and uninflamed mucosa of 3 patients with ileocolonic CD and 6 patients with UC. Eleven patients (2 colonic CD, 2 ileocolonic CD and 7 UC) were taking no drug and biopsies were collected at the time of initial diagnosis. Moreover, biopsies were taken from 18 patients (7 colonic CD, 2 ileocolic CD, and 9 UC) receiving mesalamine, 10 patients (1 colonic CD, 2 ileocolonic CD, and 7 UC) taking steroids and 6 patients (2 colonic CD, 1 ileocolonic CD, 1 ileal CD and 2 UC) on immunosuppressive drugs. In all these patients, endoscopy was performed for a clinical relapse of the disease.

Additionally, surgical specimens were taken from 15 patients with colonic CD and 19 patients with UC undergoing surgery for a chronic active disease poorly responsive to medical treatment and from 10 patients with ileal CD undergoing surgery due to stricturing disease. In 3 out of 19 UC patients, surgical specimens were available from both involved and uninvolved mucosa. Clinical characteristic of IBD patients are shown in table 1.

**Table 1:** Clinical characteristic of Crohn's disease patients and ulcerative colitis patients.

	CD n = 72	UC n = 53
***Gender, male: n (%)***	38 (52.7)	28 (52.8)
***Age: median (range)***	33 (22–65)	50 (20–82)
***CD location: n (%)***		
Terminal ileum	24 (33.3)	
Pre-anastomotic ileum	5 (6.9)	
Ileo-colon	13 (18.1)	
Colon	30 (41.7)	
***UC extent: n (%)***		
Proctitis		7 (13.3)
Left-side colitis		19 (35.8)
Extensive colitis		27 (50.9)
***Current therapy: n (%)***		
None	4 (5.5)	7 (13.3)
Sistemic CS	32 (44.4)	29 (54.7)
Budesonide	3 (4.2)	
Mesalamine	21 (29.2)	11 (20.7)
ISS	8 (11.1)	3 (5.6)
Anti-TNF	2 (2.8)	1 (1.9)
Sistemic CS+ISS	2 (2.8)	1 (1.9)
Sistemic CS+Anti-TNF		1 (1.9)

*Abbreviations:* CD, Crohn's disease, UC, ulcerative colitis, CS, corticosteroids, ISS, immunosuppressive drugs, TNF, tumor necrosis factor.

Controls (CTR) included biopsies taken from unaffected colonic mucosa of 22 subjects and unaffected ileal mucosa of 7 subjects undergoing colonoscopy for colorectal cancer screening. Additional controls were mucosal specimens taken from macroscopically and microscopically unaffected colonic areas of 24 patients undergoing surgery for colon cancer.

Autologous peripheral blood samples were obtained by standard venipucture from 9 UC patients, 27 CD patients and 9 CTR.

### RNA extraction, cDNA preparation and real-time PCR

Total RNA was extracted using TRIzol reagent (Invitrogen, Milan, Italy). A constant amount of RNA (1 µg/sample) was retro-transcribed into complementary DNA (cDNA) and then 1 µl of cDNA/sample was amplified using the following conditions: denaturation 1 minute at 95°C; annealing 30 seconds at 62°C for TNF-α and at 60°C for β-Actin, followed by 30 seconds of extension at 72°C. Primers sequence was as follows: TNF-α: forward, 5′-AGGCGGTGCTTGTTCCTCAG-3′; reverse, 5′- GGCTACAGGCTTGTCACTCG -3′; β-actin: forward, 5′-AAGATGACCCAGATCATGTTTGAGACC-3′; reverse, 5′-AGCCAGTCCAGACGCAGGAT-3′) was used as internal control gene.

CD163 was evaluated using a commercial TaqMan probe (Applied Biosystems, Foster City, CA). RNA expression was calculated relative to the housekeeping β-Actin gene on the base of the ΔΔCt algorithm.

### Total protein extraction and Western blotting

Colonic mucosal explants of CTR, UC patients and CD patients were lysed on ice in buffer containing 10 mM HEPES (pH 7.9), 10 mM KCl, 0.1 mM EDTA, 0.2 mM EGTA and 0.5% Nonidet P40 supplemented with 1 mM dithiothreitol, 10 mg/ml aprotinin, 10 mg/ml leupeptin, 1 mM phenylmethylsulfonyl fluoride, 1 mM Na3VO4 and 1 mM NaF. Lysates were clarified by centrifugation at 4°C, 12.000×g for 30 minutes, and separated on 8% sodium dodecyl sulphate-poliacrilamide gel electrophoresis. CD163 was detected using a mouse anti-human CD163 (AbD Serotec Endeavour House, Langford Lane, Kidlington, UK) followed by a horseradish peroxidase–conjugated rabbit anti-mouse IgG monoclonal antibody (Dako, Milan, Italy). The reaction was detected with a sensitive enhanced chemiluminescence kit (Pierce, Rockford, IL). After the analysis of CD163, blots were stripped and incubated with a mouse anti-human β-Actin antibody (Sigma-Aldrich, Milan, Italy) as internal loading control, followed by a goat anti-mouse antibody conjugated to horseradish peroxidase.

### Immunohistochemistry

Immunohistochemistry was performed on formalin-fixed, paraffin-embedded sections of CTR and IBD patients. The sections were deparaffinized and dehydrated through xylene and ethanol and the antigen retrieval was performed in citrate buffer (pH 6.0) for 20 minutes in microwave. Immunohistochemical staining was performed using a mouse monoclonal antibody directed against human CD163 (Biocare Medical, Concord, CA) or human CD68 (Dako) at room temperature for 1 hour followed by a biotin-free HRP-polymer detection technology with 3,3′diaminobenzidine (DAB) as a chromogen (MACH 4 Universal HRP-Polymer Kit, Biocare Medical). The sections were counterstained with haematoxylin, dehydrated and mounted. Isotype control IgG-stained sections were prepared under identical immunohistochemical conditions as described above, replacing the primary antibody with a purified mouse normal IgG control antibody (R&D Systems, Minneapolis, MN, USA). The CD163- and CD68-expressing cells were counted in at least 3 fields per section using IAS 2000 System (Delta Sistemi, Rome, Italy) and expressed as number of cells for high power field (hpf).

### *Ex vivo* organ cultures

Freshly obtained normal colonic mucosal samples were cultured as described elsewhere [26]. Briefly, samples were placed on iron grids with the mucosal face upward in the central well of an organ culture dish containing AQIX medium (Aqix Ltd, London, UK) supplemented with 1% L-glutamine penicillin (P) (100 U/ml), streptomycin (S) (100 µg/ml) and gentamycin (G) (50 µg/ml) (all from Lonza Vervies, Belgium) in the presence or absence of TNF-α (20 ng/ml, R&D Systems) or IL-6 (50 ng/ml, R&D Systems). Dishes were then placed in a tight container with 95% O2, and 5% CO2 at 37°C, at 1 bar. After 24 hours, mucosal samples were used to evaluate CD163 expression by Western blotting.

### Cell Isolation, Purification of HLA-DR-expressing LPMC and Culture

Human peripheral blood mononuclear cells (PBMC) were isolated from EDTA-stabilized blood samples of CTR and IBD patients by Ficoll gradients and used for flow cytometry analysis or to perform cell cultures in RPMI 1640 medium supplemented with 10% fetal bovine serum (FBS) and P/S/G.

LPMC were isolated from colonic specimens of CTR and IBD patients as described elsewhere [27] and resuspended in RPMI 1640 medium supplemented with 10% FBS and P/S/G. Moreover, LPMC isolated from surgical specimens of CTR and CD patients were depleted of CD3- e CD19-positive cells and then used to purify HLA-DRII-expressing cells by commercial kits (Miltenyi Biotec, Bergish Gladbach, Germany) according to the manufacturer's instruction. To evaluate whether inflammatory stimuli can modulate CD163 expression, normal LPMC (or HLD-DR-expressing LPMC) were seeded at a concentration of 2×10^6^ cells/ml into 24-well culture dishes either left unstimulated or stimulated with TNF-α 20 ng/ml, R&D Systems) or IL-6 (50 ng/ml, R&D Systems) for 48 hours and then analyzed by Western blotting. To examine functional properties of CD163, IBD LPMC (or IBD HLA-DR-expressing LPMC) and PBMC were seeded at a concentration of 1×10^6^ cells/ml into 48-well culture dishes pre-coated with 10 µg/ml EDHU1-Ab (AbD Serotec, Düsseldorf, Germany) or control Ab mouse IgG1 (R&D Systems) for 2 hours at 37°C and cultured for 6 and 48 hours. To examine whether CD163 expression correlates with TNF-α production, PBMC were isolated from IBD patients and used to purify CD14+ monocytes using isolation kit (Miltenyi Biotec, Bergish Gladbach, Germany) according to the manufacturer's instruction. RNA was then extracted from those cell samples and CD163 and TNF-α RNA transcripts were evaluated by Real Time PCR.

### TNF-α enzyme-linked immunosorbent assay

TNF-α was measured in supernatants of IBD LPMC and PBMC cultured for 48 hours as described above using a sensitive commercial enzyme-linked immunosorbent assay (ELISA) kit (R&D Systems) according to the manufacturer's instructions.

### Flow cytometry analysis

CTR and IBD PBMC were stained with CD163 PE (1∶50 final dilution; eBioscience, San Diego, CA), CD14 FITC, (1∶50 final dilution, Immunotools, Friesoythe; Germany), CD16 PerCP (1∶50 final dilution; Invitrogen) or isotype control IgGs (BD Biosciences) and cell-surface fluorescence intensity was assessed using a FACSCalibur analyzer and analyzed using CellQuest software (BD Biosciences, Milan, Italy).

### Statistical analysis

Differences between groups were compared using the Mann–Whitney U test and Wilcoxon test. Correlation between CD163 and TNF-α was examined using Spearman's non parametric correlation.

## Results

### CD163 RNA transcripts are up-regulated in IBD

**Figure 1:**
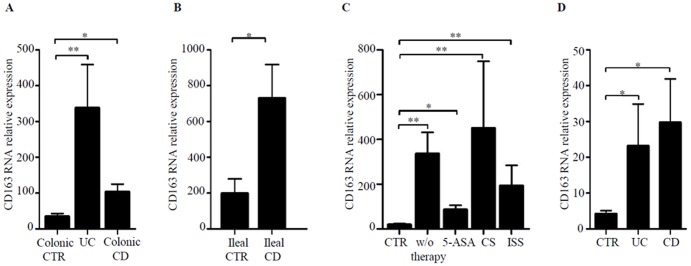
CD163 transcripts are increased in inflamed IBD mucosa. A. CD163 RNA expression was evaluated in colonic biopsies taken from 22 normal controls (CTR), 25 patients with Ulcerative Colitis (UC) and 19 patients with Crohn's disease (CD) by real-time PCR and levels were normalized to β-actin. Data indicate mean ± SEM of all samples; *p = 0.01; **p<0.0001. B. CD163 RNA expression was evaluated in ileal biopsies taken from 7 ileal CTR and 6 ileal CD patients by real-time PCR. Data indicate mean ± SEM of all samples; *p = 0.03. C. Colonic biopsies taken from 22 CTR, 11 IBD patients (4 CD and 7 UC) receiving no therapy (w/o therapy), 18 IBD patients (9 CD and 9 UC) treated with mesalamine (5-ASA), 10 IBD patients (3 CD and 7 UC) treated with steroids (CS) and 5 IBD patients (3 CD and 2 UC) treated with immunomodulators (ISS) were analyzed for CD163 RNA expression by real-Time PCR. Levels are normalized to β-actin. Data are expressed as mean ± SEM of all samples; *p = 0.01; **p<0.01. D. CD163 RNA expression was evaluated in LPMC from 6 normal controls (CTR), 4 patients with UC and 6 patients with CD by real-time PCR. Levels are normalized to β-actin. Data indicate mean ± SEM of all samples; *p = 0.02.

CD163 RNA expression was more pronounced in both CD and UC colonic samples in comparison to CTR (Fig. 1A). UC biopsies contained more CD163 transcripts than CD biopsies but the difference was not statistically significant (Fig. 1A). High CD163 RNA was also seen in ileal CD samples as compared to ileal CTR samples (Fig. 1B). To exclude the possibility that up-regulation of CD163 in IBD was secondary to current therapy, CD163 RNA transcripts were compared between patients receiving or not drugs. Figure 1C shows that CD163 RNA expression was not influenced by the ongoing treatments. Finally, we showed that CD163 transcripts were up-regulated in IBD LPMC as compared to control samples (Fig. 1D).

### CD163-positive cells accumulate in the inflamed intestine of IBD patients

**Figure 2:**
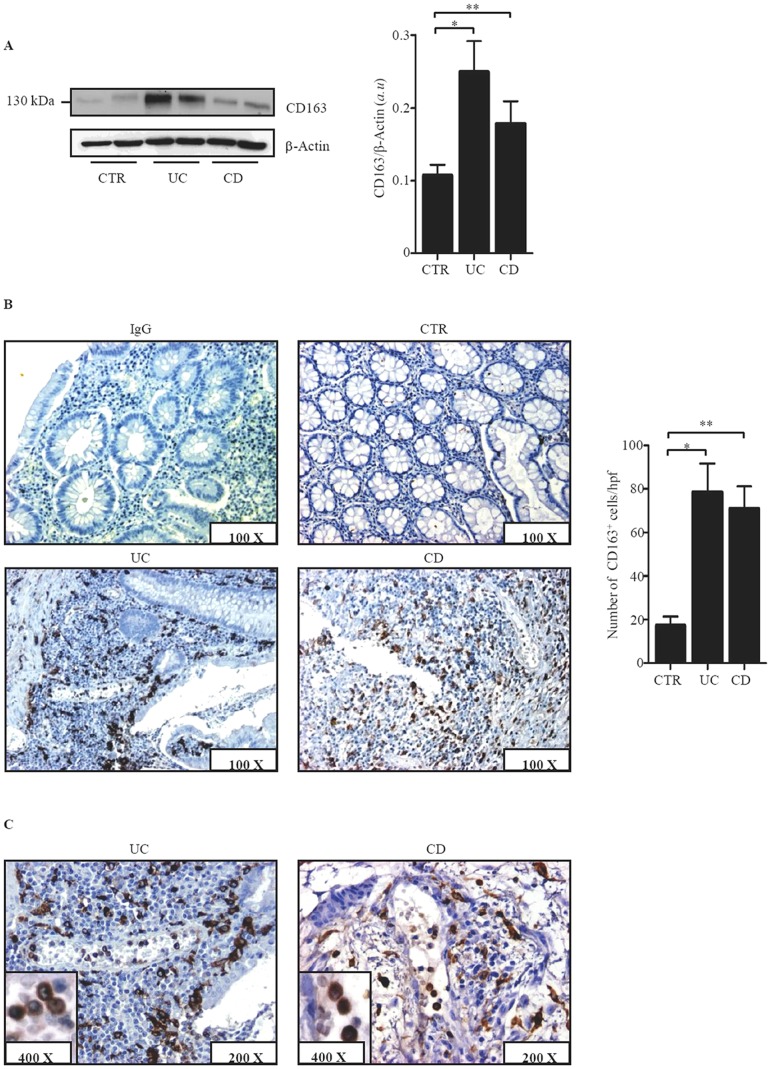
CD163 protein expression is increased in IBD. A. Representative Western blots showing CD163 and β actin in total proteins extracted from mucosal samples of 2 CTR, 2 UC patients and 2 CD patients. Right panel shows the quantitative analysis of CD163/β-actin ratio in mucosal samples taken from 8 CTR, 8 UC patients and 8 CD patients as measured by densitometry scanning of Western blots. Values are expressed in arbitrary units (a.u.) and indicate mean ± SEM of all samples; *p = 0.003; **p = 0.03. B. Representative photomicrographs (100× original magnification) of CD163-stained paraffin-embedded sections of surgical samples taken from 1 CTR, 1 patient with UC and 1 patient with CD. Isotype control antibody-stained section is also shown. Right panel shows the number of CD163-positive cells for high power field (hpf) in colonic sections taken from 3 CTR, 3 UC patients and 4 CD patients. Data are expressed as mean ± SD; *p = 0.03; **p = 0.02. C Representative photomicrographs (200× original magnification) of CD163-stained paraffin-embedded sections of surgical samples taken from 1 patient with UC and 1 patient with CD. CD163-positive cells are evident inside and around vessels. Insets show higher magnification (400×) images.

Western blotting analysis showed that CD163 was expressed in all IBD and CTR samples, but immunoreactivity corresponding to CD163 was more pronounced in IBD in comparison to CTR (Fig. 2A). Immunohistochemical analysis confirmed the abundant expression of CD163 in IBD and showed accumulation of these cells around the deep ulcers (Fig. 2B, right lower panel) and around and inside the blood vessels (Fig. 2C).

**Figure 3:**
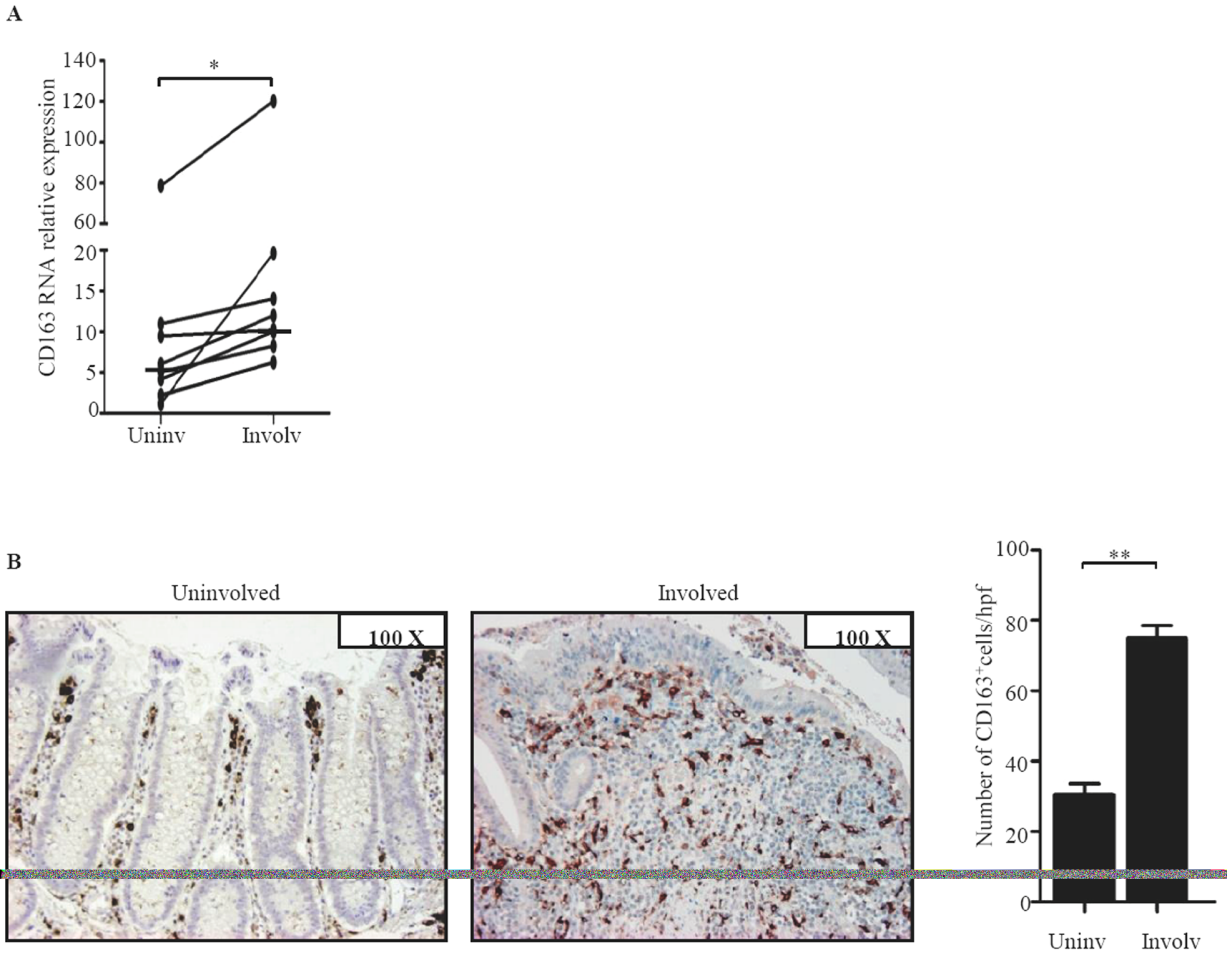
CD163 RNA and protein expression is increased in the inflamed areas of IBD. A. Paired biopsies taken from the involved (Involv) and uninvolved (Uninv) mucosa of 6 UC patients and 3 CD patients were analyzed for CD163 RNA expression by real-time PCR. Levels are normalized to β-actin; horizontal bars indicate the median values; *p = 0.003. B. Representative photomicrographs (original magnification 100×) of CD163-stained sections of colonic mucosal samples taken from involved and uninvolved mucosa of 1 UC patient. Right panel shows the number of CD163-positive cells per high power field (hpf) of colonic sections taken from the involved (Involv) and uninvolved (Uninv) mucosa of 3 UC patients. Data indicate the mean values ± SEM of all samples; **p = 0.01.

**Figure 4:**
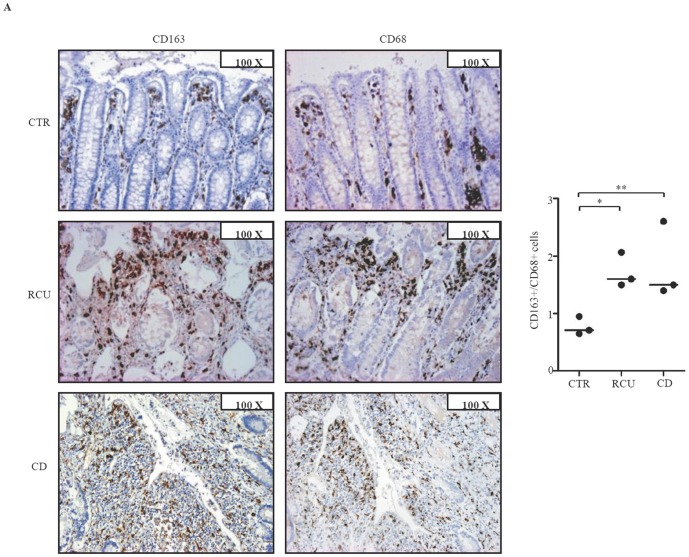
Increased CD163+cells/CD68+ cells ratio in IBD tissue. A. Representative photomicrographs (100× original magnification) in serial paraffin-embedded sections of surgical samples taken from 1 CTR, 1 patient with UC and 1 patient with CD and stained with CD163 or CD68. Right panel shows the ratio of CD163+ and CD68+ cells counted in colonic sections taken from 3 CTR, 3 UC patients and 3 CD patients. Horizontal bars indicate the median values; *p = 0.03; **p = 0.02.

To examine whether, in IBD, CD163 is restricted to areas with mucosal lesions, paired biopsies were taken from both involved and uninvolved areas of IBD patients and examined for CD163 by real-time PCR and immunohistochemistry. CD163 RNA and protein expression was up-regulated in the inflamed samples as compared to uninvolved mucosal samples of the same IBD patients (Fig. 3). In contrast, expression of CD163 did not differ between uninvolved biopsies of IBD patients and normal CTR (not shown). Moreover, serial sections of IBD and control specimens stained with CD163 or CD68 showed that the number of CD68-expressing cells was higher than that of CD163-positive cells in control samples, while in both CD and UC the number of CD163-positive cells exceeded that of CD68-expressing macrophages (Fig. 4). These data suggest that up-regulation of CD163 cells in IBD mucosa is not secondary to the increased macrophage infiltration.

### CD163 -positive PBMC are increased in IBD

**Figure 5:**
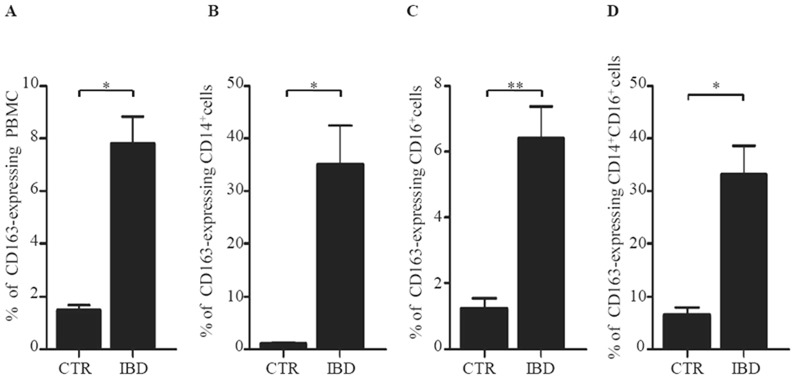
CD163-expressing cells are increased in the blood of IBD patients. A. The Histograms show the percentage of CD163-expressing cells in total PBMC (A), CD14^+^ cells (B), CD16^+^ cells (C) and CD14^+^ CD16^+^ cells (D) of 23 IBD patients (5 UC patients, 18 CD patients) and 9 CTR. Cells were isolated from the blood of IBD patients and controls and examined by flow-cytometry as indicated in material and methods. Data indicate the mean values ± SEM of all samples; *p<0.001 **p = 0.001.

The fact that, in IBD tissue, CD163-expressing cells are located around and inside blood vessels (Fig. 2B–C) raises the possibility that these cells are in part recruited from the systemic circulation. Thus, in subsequent experiments, we evaluated the expression of CD163 in PBMC of IBD patients and CTR. The percentage of CD163-expressing PBMC was increased in IBD patients as compared to CTR (Fig. 5A). Further analysis revealed that the fractions of CD14+, CD16+ and CD14+/CD16+ cells expressing CD163 were increased in IBD PBMC in comparison to control PBMC (Fig. 5 B–D).

### IL-6 enhances CD163 expression in normal colonic explants and LPMC

**Figure 6:**
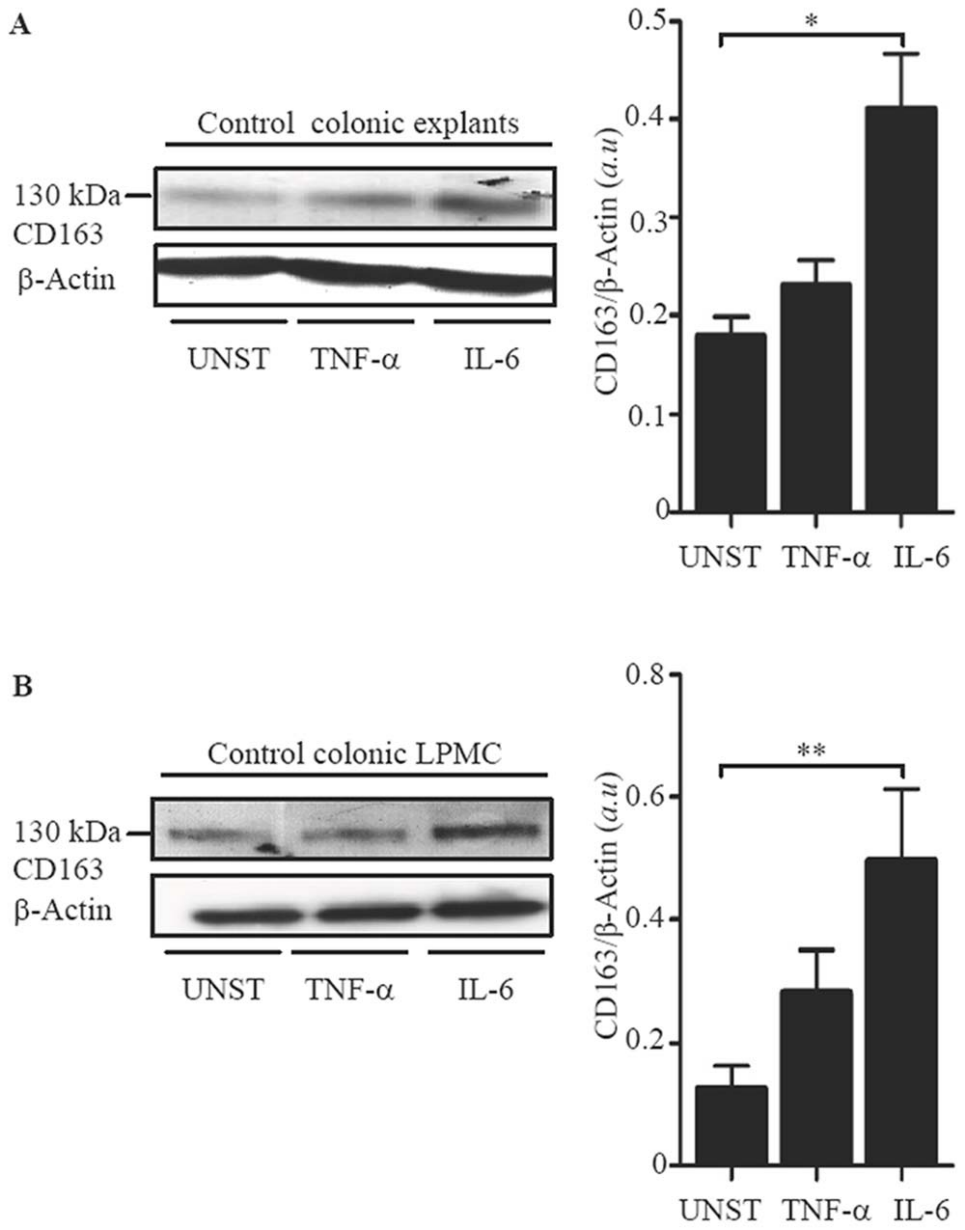
IL-6 enhances CD163 expressions in normal colonic explants and LPMC. A. Representative Western blots showing CD163 and β-actin in total proteins extracted from normal colonic explants treated with or without (unstimulated = UNST) TNF-α (20 ng/ml) or IL-6 (50 ng/ml) for 24 hours. Right panel shows the quantitative analysis of CD163/β-actin ratio in normal colonic explants. Values are expressed in arbitrary units (a.u.) and indicate mean ± SEM of four separate experiments; *p = 0.04. B. Representative Western blots showing CD163 and β-actin in total proteins extracted from normal LPMC treated as above for 48 hours. Right panel shows the quantitative analysis of CD163/β-actin ratio in LPMC protein extracts. Values are expressed in arbitrary units (a.u.) and indicate mean ± SEM of three separate experiments; **p = 0.01.

Next, we determined whether CD163 can be modulated by cytokines over-produced in IBD [28], [29]. To this end, normal colonic explants and LPMC were stimulated with TNF-α and IL-6, two cytokines that up-regulate CD163 in other systems [28], [29]. Western blot analysis showed that IL-6, but not by TNF-α increased CD163 protein expression in both explants and LPMC (Fig. 6A–B). To examine whether increased expression of CD163 resulted from direct effects of IL-6 on normal intestinal macrophages, HLA-DR-expressing CD3- and CD19-negative LPMC were stimulated with IL-6 for 6 hours and then CD163 RNA was evaluated by real-time PCR. IL-6 enhanced CD163 RNA transcripts in all samples analyzed (1.2±0.2 in unstimulated cells vs 5±1.4 in IL-6-stimulated cells).

### Cross-linking of CD163 with EDHU1-Ab increases TNF-α production

**Figure 7:**
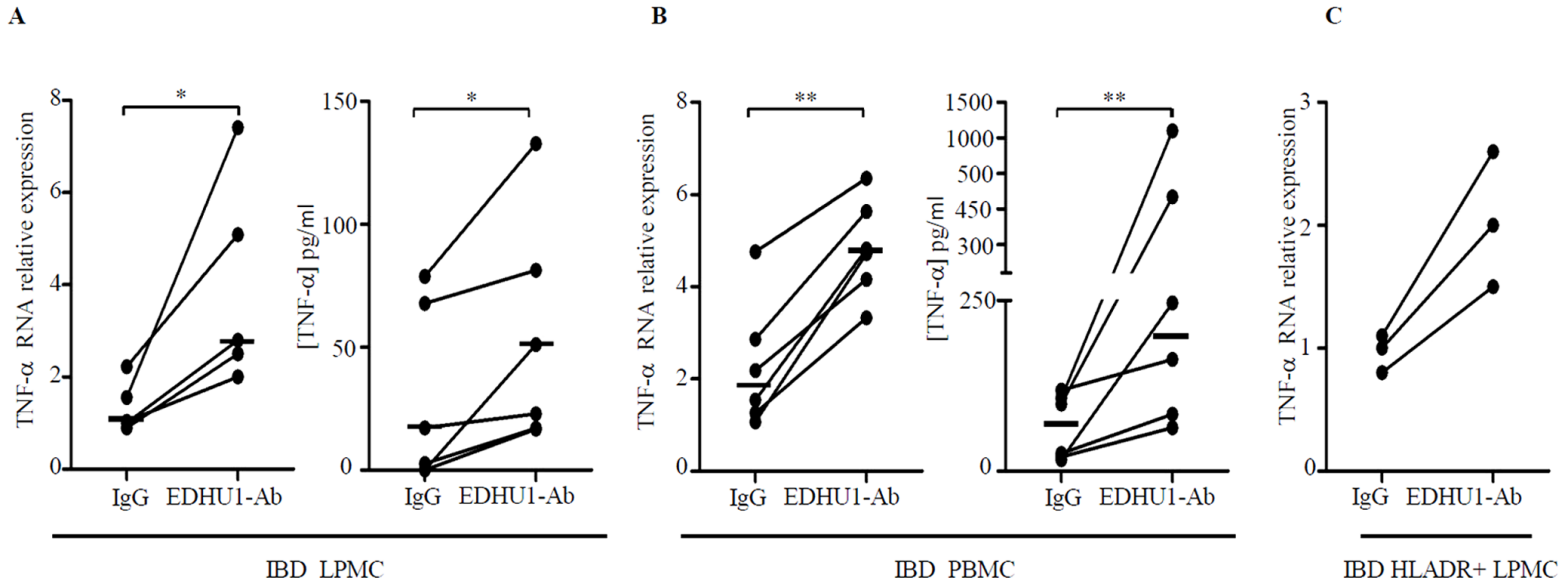
Cross-linking of CD163 with the EDHU1-Ab enhances TNF-α expression in LPMC, PBMC and in purified HLADR-expressing LPMC of IBD patients. A–B. Left panels. TNF-α RNA expression was evaluated in LPMC of 4 CD patients and 1 UC patients (A) and in PBMC of 3 CD patients and 3 UC patients (B) treated with EDHU1-Ab or control IgG for 6 hours by real-time PCR. Levels are normalized to β-actin; horizontal bars indicate the median values; *p = 0.03; **p = 0.01. Right panels. TNF-α secretion was measured in supernatants of IBD LPMC and PBMC treated as described above for 48 hours. Horizontal bars indicate the median values; *p = 0.03; **p = 0.01. C. TNF-α RNA expression was evaluated by real-time PCR in HLA-DR-expressing, CD3- and CD19-negative LPMC of 3 patients with CD and treated with EDHU1-Ab or control IgG for 6 hours. Levels are normalized to β-actin.

In a final set of experiments we evaluated if CD163-delivered signals control cytokine expression. IBD LPMC and PBMC were cultured in the presence or absence of a specific activating CD163 monoclonal antibody (EDHU1-Ab) or control IgG for 6–48 hours and TNF-α RNA and protein were then examined by real-time PCR and ELISA respectively. Cross-linking of CD163 with EDHU1-Ab increased TNF-α RNA and protein expression and this was evident in both LPMC and PBMC (Fig. 7A–B). Consistently, stimulation of CD HLA-DR-expressing CD3- and CD19-negative LPMC with EDHU1-Ab increased TNF-α RNA expression (Fig. 7C). Although these data indicate that CD163 cross-linking on monocytes/macrophages enhances TNF-α expression, no correlation was seen between CD163 and TNF-α transcripts in CD14+ monocytes isolated from blood of IBD patients (not shown), perhaps due to the multiple signals controlling TNF-α production in monocytes/macrophages.

## Discussion

The present study was undertaken to evaluate the expression and function of CD163 in IBD. CD163 is a multifunctional receptor, which can bind multiple ligands and activate various intracellular pathways, thereby controlling many biological processes. Initial studies showed that CD163 identifies a specific subsets of alternatively activated macrophages [30], which exert anti-inflammatory effects following interaction and internalization of the complex Hb-Hp [10]. This function of CD163 was supported by the demonstration that CD163-expressing macrophages constitute the predominant macrophage population during the late or resolution phase of many inflammatory reactions [31], [32] and CD163 expression is strongly induced by anti-inflammatory mediators, such as glucocorticoids and IL-10 [33], [34]. More recently, it has been demonstrated that CD163 can also bind both Gram-negative and Gram-positive bacteria and activate a number of intracellular events, which lead to production of pro-inflammatory cytokines [14]. Therefore, it is plausible that CD163 may have a dual role in the control of immune responses perhaps depending on the microenvironment in which CD163-expressing cells are located.

We here show that IBD-related inflammation is marked by enhanced CD163 expression at both RNA and protein level. High CD163 is restricted to mucosal areas with active inflammation and not influenced by current therapy. While this study was ongoing, Bain and co-workers showed that CD14-positive LPMC isolated from the inflamed ileum of CD patients express low levels of CD163 as compared to control cells [25]. We have attempted to reproduce such results using the same LPMC isolation and flow-cytometry procedures adopted by Bain. However, CD163 was undetectable on LPMC of both IBD patients and controls using 3 different commercial flow-cytometry antibodies, including that used by Bain and co-workers. The reason for this apparent discrepancy remains unknown. The fact that analysis of this receptor in our samples was performed using a very sensitive technique (i.e. real-time PCR) and data were confirmed by Western blotting and immunohistochemistry suggests however that CD163 is over-expressed in the inflamed mucosa of IBD patients. This hypothesis is supported by a previous immunostaining study showing enhanced accumulation of CD163+ macrophages in the intestinal mucosa of patients with CD and patients with spondyloarthropathy [23]. CD163 was detectable in PBMC by flow-cytometry and positivity was not affected by exposure of cells to reagents used to isolate LPMC (e.g. EDTA, collagenase). Thus, it is unlikely the difficulty to detect CD163 in LPMC by flow-cytometry is secondary to cleavage of the receptor during the cell preparations.

The relevance of the increased number of CD163+ cells in IBD tissue is highlighted by further observations. First, the increase of CD163+ cells reflects an increase of a specific macrophage subpopulation rather than a global increase of macrophages, since the number of CD68+ cells was similar or even decreased to that of CD163+ cells in IBD samples. Second, the up-regulation of CD163+ cells in patients with CD and patients with UC could reflect a similar pathophysiological mechanism, which accounts for the accumulation of this particular cell subset in the two IBD. Third, CD163 was enhanced in normal LPMC by IL-6, a cytokine that is over-produced in both CD and UC and supposed play a key role in the pathogenesis of IBD-associated tissue-damaging immune response. Fourth, cross-linking of CD163 with a specific activating antibody leads to enhanced production of TNF-α in cultures of IBD LPMC and HLADR-expressing LPMC.

Overall our data are in line with previous studies documenting high expression of CD163 in pathological conditions. For example, Yawalkar and Fuentes-Duculan demonstrated that CD163-positive macrophages infiltrate heavily the lesional skin of psoriatic patients and express bioactive forms of pro-inflammatory cytokines, such IL-12 and IL-23 [15], [16]. Consistent with this is the demonstration that CD163-positive macrophages infiltrating the fibrous tissue in facet joints of patients with ankylosing spondylitis are a major source of inflammatory cytokines, such IL-23 [35]. Wakusawa et al described a patient with Necrobiosis lipoidica associated with Insulin-dependent type 1 diabetes mellitus, in which granulomas contained mostly pro-inflammatory CD163-positive macrophages [36]. Increased expression of CD163 has been also seen in skin macrophages and peripheral blood monocytes of patients with systemic sclerosis, an autoimmune disease characterized by local inflammatory infiltrates and widespread fibrosis [37]. CD163-expressing macrophages are also overrepresented in the colon of patients with diverticulitis [38] and in the kidney of patients with IgA nephropathy [39].

In conclusion, data of the present study show that CD163+ cells are abundant in the inflamed gut of patients with IBD and suggest a role for these cells in the amplification and perpetuation of the ongoing mucosal inflammation in IBD.
